# Diversity of root-knot nematodes in Moroccan olive nurseries and orchards: does *Meloidogyne javanica* disperse according to invasion processes?

**DOI:** 10.1186/s12898-017-0153-9

**Published:** 2017-12-19

**Authors:** Mohamed Aït Hamza, Nadine Ali, Johannes Tavoillot, Odile Fossati-Gaschignard, Hassan Boubaker, Abdelhamid El Mousadik, Thierry Mateille

**Affiliations:** 1 Faculté Des Sciences, Laboratoire LBVRN, Université Ibn Zohr, BP 8106, 80000 Agadir, Morocco; 2 IRD, UMR, CBGP, 755 Avenue du Campus Agropolis, CS30016, 34988 Montferrier-sur-Lez Cedex, France; 3 Faculty of Agriculture, Plant Protection Department, Tishreen University, PO Box 230, Latakia, Syrian Arab Republic; 4 Faculté Des Sciences, Laboratoire LBMPV, Université Ibn Zohr, BP 8106, 80000 Agadir, Morocco

**Keywords:** Distribution, Diversity, Invasion, *Meloidogyne*, Morocco, Nursery, Olive, Orchard, Species identification

## Abstract

**Background:**

Root-knot nematodes (RKN) are major pest of olive tree (*Olea europaea* ssp. *europaea*), especially in nurseries and high-density orchards. Soil samples were collected from main olive growing areas of Morocco, to characterize *Meloidogyne* species and to discuss the contribution of biotic and abiotic factors in their spatial distribution.

**Results:**

RKN were found in 159 soil samples out of 305 from nurseries (52.1% occurrence) and in 11 out of 49 soil samples from orchards (23.2% occurrence). Biochemical and molecular characterisation (PAGE esterase and SCAR) revealed the dominance of *M. javanica* both in nurseries and orchards with minor presence of *M. incognita* only in nurseries, and *M. arenaria* in only one nursery. RKN were distributed on aggregated basis. Frequent presence of *M. javanica* in orchards might have come from nurseries. In contrast, the detection of *M. incognita* in nurseries alone suggests that this species could not reproduce in orchards because of either the competition with other plant-parasitic nematodes or unfit local habitats. The impact of environmental variables (climate, habitat origin and physicochemical characteristics of the substrates) on the distribution of *Meloidogyne* species is also discussed.

**Conclusion:**

Olive nurseries in Morocco are not able to guarantee the safety of rooted plants. As a result, olive production systems are exposed to strong RKN invasion risks. Consequently, the use of healthy substrates in nurseries may prevent plant-parasitic nematode induction in orchards.

## Background

Sustainable management of key taxa depends upon understanding their distribution and behaviour towards biotic and abiotic factors. Studying the factors of RKN population dispersal can facilitate to understand their spatial structure [1]. Ecologists and conservation managers depend on spatial models to assess environmental effects on distribution of a species. These models facilitate to develop reserve selection and survey design to manage various species.

Distribution models are categorized into two groups: (1) some simulate interactive processes between environment and organisms, (2) others use pattern analysis to reveal correlation among target taxa and environmental variables. Biological (capacity of an organism to disperse and reproduce), physical (mountains or oceans) and environmental (soil texture, moisture conditions) factors hinder species dispersal [2]. These models require detailed information about organism and environment over a period of time to predict spatial and temporal patterns of an organism [3].

The production of commercial olive plantlets in the Mediterranean basin, especially in Morocco, provides a favourable environment for the development of plant pests [4]. The geographic location of Morocco, compared to other Mediterranean countries, offers specific orography and bio-climates, with endemic vegetation [5]. High mountain ranges (exceeding 4000 m in altitude) create a complex and highly compartmentalized structure, with extensive plateaux and plains. Climate of the country is deeply influenced by the Atlantic Ocean with annual rainfall of 30–2000 mm.

In Morocco, nursery substrates are often prepared with soil from cropped fields or natural environments that could be potentially infested with soil-borne pathogens such as *Verticillium dahliae* Kleb. (i.e., *Verticillium wilt*) [5] and plant-parasitic nematodes (PPN). The use of pathogen-free planting materials and non-infested soils is necessary during the early years of olive cultivation. The threat of these pathogens to olive production has also been recognised by the European Union [6].

PPN are microscopic, round and filiform worms living in soil and/or inside plant root tissues. They generally parasite the underground parts (roots, tubers, rhizomes) of the plants. They cause significant agricultural damage in the world (about 14% yield loss), that reaches over $100 billion per year [7]. Abiotic factors such as pH, soil type, organic matter content, moisture [8], and local climatic conditions affect PPN development [9]. They move only short distances and, thus their dissemination is via water [10] and wind [11]. Human activities such as the introduction of infected planting material or diffusion of infested soil with nursery practices also contribute to spreading [12]. *Meloidogyne* spp. are major PPN, causing a worldwide loss of about 50 billion Euros [13, 14]. *Meloidogyne* species like *M. javanica, M. inconita, M. arenaria, M. hapla* and *M. lusitanica* are known to infect olive trees [15, 16]. Recently *M. baetica* and *M. spartelensis* were also identified on wild olive trees in southern Spain and northern Morocco [17]. Nevertheless, little information is available about PPN host-parasite relationship between RKN and olive plantlets. RKN are known as major pest of olive trees, especially in nurseries having favourable irrigation conditions [18]. Experiments have demonstrated effect of RKN on olive plant growth and of susceptible olive cultivars [19].

RKN control is a challenging task, because: (i) their occurrence is worldwide especially under hot climate; (ii) they are highly diversified; and (iii) they exhibit various reproduction methods (mitotic and meiotic parthenogenicity and amphimixis) [20]. Therefore, in order to manage their infestation, identification of *Meloidogyne* species is basic requirement to understand its ecology, physiology and reproduction [21]. Conventional methods for RKN identification based on morphological traits require a great deal of skill and are often inconclusive. Polyacrylamide gel electrophoresis (PAGE) isozyme analysis is a relatively fast way to identify *Meloidogyne* species [22]. However, isozyme analysis can only be done with mature females embedded in roots, and not with second-stage juveniles or eggs in the soil. SCAR (Sequence Characterized Amplified Region) based molecular biomarkers are used to confirm *Meloidogyne* species [23].

Previous surveys revealed scarce populations of *M. arenaria*, *M. hapla* and *M. spartelensis* in wild olive whereas *M. javanica* dominated in cultivated areas of Morocco [16]. We hypothesized that its widespread distribution might be due to inductions from nurseries. Therefore, objective of this study is to test the hypothesis by: (i) characterizing the *Meloidogyne* species in nurseries and orchards of main olive-producing areas of Morocco; (ii) analysing their distribution in nurseries, impact of the climate, habitat and physicochemical characteristics of the substrates; and (iii) discussing their introduction from nurseries to orchards, especially in relevance to *M. javanica*.

## Methods

### Site description

**Fig. 1:**
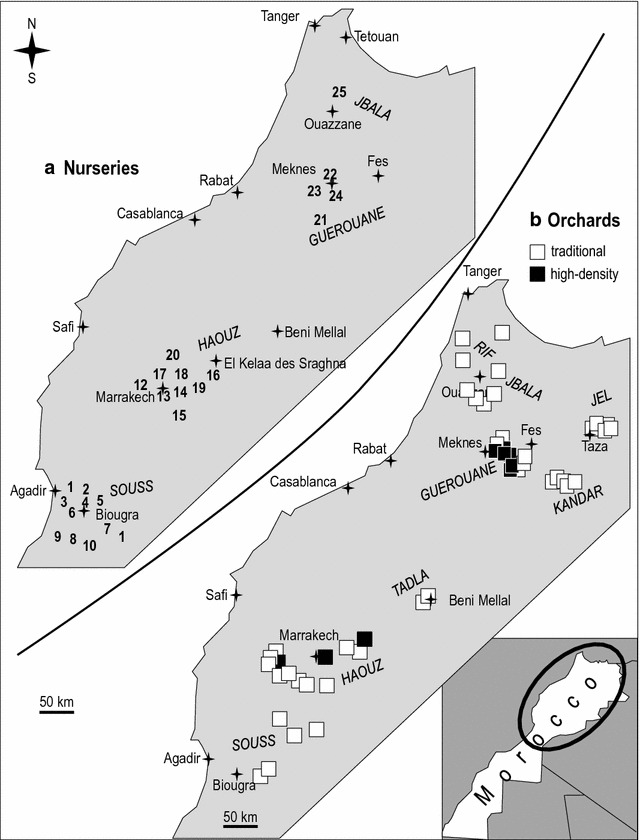
Distribution of the olive nurseries (**a**) and orchards (**b**) surveyed in Morocco

Surveys were conducted in the main olive (*Olea europaea* subsp. *europaea*) cultivated areas of Morocco (Fig. 1) ranging from the Strait of Gibraltar in the north to Agadir in the south, covering various soil types. Annual precipitation in the area ranges between 200 and 1000 mm from south to north, temperatures between 5 and 45 °C and altitudes from 200 to 1700 m [24]: (i) in the Jbala region along the west side of Rif Mountains in the north; (ii) near Taza in the Jel Plains of eastern Morocco; (iii) near Fes in the Kandar region located in northern Middle Atlas Mountains; (iv) south of Meknes in the Guerouane region; (v) near Beni Mellal in the Tadla region located on the north side of the southern Middle Atlas Mountains; (vi) near Marrakech in the Haouz region, on the north side of the High Atlas Mountains; and (vii) near Agadir in the Souss region located on the south side of the High Atlas Mountains. Overall twenty-five nurseries were selected for (i) production and the diversity of the varieties, (ii) diversity of culturing substrates, and (iii) geographic distribution (Fig. 1a; Table 1). Forty-nine orchards were selected according to traditional (100 trees/ha; no irrigation) and high-density (up to 2200 trees/ha; drip irrigation) orchards [25] (Fig. 1b; Table 1).

**Table 1:** Location and characteristics of the olive nurseries and orchards surveyed in Morocco

Location		Nurseries				Orchards		
Geographic region	City	No. of nurseries	Main habitat origin of the substrates	Olive cultivar	No. of samples	No. of sites	Growing system	Olive cultivar
Rif	Asilah					1	Traditional	Picholine marocaine
	Chefchaouen					2	Traditional	Picholine marocaine
Jbala	Ouazzane	1	Clay marls, sand, forest soil and topsoil	Picholine marocaine	5	5	Traditional	Picholine marocaine
				Haouzia	5			
				Menara	5			
Guerouane	Meknes	4	Yellow sand, topsoil, mature manure and local compost	Picholine marocaine	15	4	High density	Picholine marocaine
				Haouzia	20	3	Traditional	Picholine marocaine
				Menara	20			
				Arbequina	5			
				Arbosana	5			
				Picual	10			
				Picholine Languedoc	5			
	El Hajeb					2	High density	Picholine marocaine
						2	Traditional	Picholine marocaine
Kandar	Sefrou					5	Traditional	Picholine marocaine
Jel	Taza					3	Traditional	Picholine marocaine
	Msoun					2	Traditional	Picholine marocaine
Tadla	Beni Mellal					1	Traditional	Picholine marocaine
Haouz	Marrakech	5	Clay marls, sand, forest soil, mountain soil and topsoil	Picholine marocaine	25	7	Traditional	Picholine marocaine
				Arbequina	10	2	High density	Arbequina + Picual + Koroneiki
				Haouzia	25			
				Menara	25			
				Picholine Languedoc	10			
				Arbosana	5			
	El Kelaa des Sraghna	3	Forest soil and topsoil	Picholine marocaine	15	1	Traditional	Picholine marocaine
				Picholine Languedoc	15			
				Menara	10			
				Haouzia	10			
	Sidi Abdellah Ghiat	1	Soil, clay and sand	Picholine marocaine	5			
	Rehamma					1	High density	Picholine marocaine
	Tammelalt					1	Traditional	Picholine marocaine
	Sidi Bou Othmane					1	Traditional	Picholine marocaine
	Tahannaout					1	Traditional	Picholine marocaine
Souss	Agadir	8	Sand, topsoil and peat moss	Picholine marocaine	15			
				Haouzia	20			
				Menara	5			
	Khmiss Aït Amira	2	Topsoil, peat, manure	Picholine marocaine	10			
	Biougra	1	Peat, soil and perlite	Menara	5			
	Taroudant					2	Traditional	Picholine marocaine
	Ouled Taima					1	Traditional	Picholine marocaine
	Ouled Berhil					1	Traditional	Picholine marocaine
	Aoulouz					1	Traditional	Picholine marocaine

### Soil sampling

In nurseries, olive plantlets are grown in 2–3-l plastic bags containing solid substrates from different origins (alluvial sandy soils, forest soils, loamy open-field soils) with different proportions of sand, peat fertilizer and animal manure. Five olive plantlets were sampled from each nursery for each variety. Information of variety, origin and substrates was recorded for each sample. In total, 305 olive plantlets were taken to the laboratory and maintained under greenhouse conditions.

In orchards, only soil samples were collected, as PPN spend a part of their life cycle in it. Samples were collected from upper rhizosphere of soil under the foliage. In each orchard, four trees at 10 m distance along transects, were chosen and from each tree five sub-samples were taken. Considering that the cultivation activities could homogenize the distribution of nematodes, twenty sub-samples were pooled into one (1-dm^3^) reference sample per orchard.

### Root-knot nematode extraction and culture

From both, nursery olive plantlets and orchard soil samples, nematodes were extracted from a 250-cm^3^ soil aliquot according to elutriation procedure [26]. RKN were identified according to genus, counted and expressed per dm^3^ of fresh soil. Susceptible tomato variety (cv. Roma) was grown in 500-cm^3^ soil of each sample under greenhouse (12 h light at 25 °C, 12 h dark at 20 °C) to multiply the populations. Presence of RKN galls and egg masses was observed after 60 days of tomato transplantation.

### Identification of *Meloidogyne* species

#### Isozyme phenotype analysis

Tomato roots were lightly washed and adult females were collected using forceps and transfer needles. 25 females and their eggs were collected per sample. Females were individually crushed in 250-µL micro-tubes containing 5 µL of Trugdill buffer with 20% sucrose (pH 8.0) [27], and stored at − 20 °C. Females of pure *M. javanica* population were prepared as above and used as the reference population. Micro-tubes were centrifuged (9500 rpm for 10 min) and 0.01% bromophenol-blue was added. Supernatants were transferred to 70 × 80 × 0.5 mm separating (7% bis-acrylamide, pH 8.4) and stacking (3.5% bis-acrylamide, pH 6.7) gels [22] whereas PAGE was processed in a Mini Protean II electrophoresis unit (BioRad^®^) at 7 °C. Each gel included two reference *M. javanica* females. Gels were incubated with α-naphthyl acetate and Fast Blue (37 °C for 1 h) to reveal Esterase (Est) phenotype bands. The band stain was fixed by placing gels in 10% acetic acid for several hours and sandwiched between cellophane sheets to dry for 48 h [28]. Est phenotype patterns were identified and labelled by bands (Rm) in reference to *M. javanica*.

#### Molecular identification

Female egg masses of Est analyses were individually incubated in distilled water for hatching. 5–10 juveniles were taken/egg mass to extract DNA. QIAGEN DNeasy Blood & Tissue kits were used for nematode DNA extraction, and PCR-SCAR assays were carried out with specific primers: OPA-12 Fare/Rare (for *M. arenaria*), OPB-06 Finc/Rinc (for *M. incognita*), and OPA-01 Fjav/Rjav (for *M. javanica*) [23]. PCR amplifications were performed in 2 µL (10 ng) of template DNA, 5 µL of PCR QIAGEN kits (Multiplex-PCR), 1 µL of each SCAR primer and 2 µL of sterile water using the GeneAmpR PCR System 9700 (Applied Biosystems^®^). PCR amplification was carried out at: initial denaturation (95 °C for 15 min), 40 cycles denaturation (94 °C for 30 s), annealing (58 °C for 90 s), elongation (72 °C for 90 s), final extension (72 °C for 10 min). Amplified products were confirmed on 1.5% agarose gel with DNA Ladder (200–10,000 pb).

### Soil and climate data recovered in nurseries

A 100-cm^3^ dry and sieved (2 mm) aliquot from each soil sample was used for physicochemical analyses in Soil Laboratory of the “Institut Agronomique et Vétérinaire Hassan II” (Agadir, Morocco). Soil texture analysis including clay (0–2 µm), fine (2–20 µm) and coarse (20–50 µm) silt, fine (50–200 µm) and coarse (200–2000 µm) sand was performed according to Stoke’s Law sedimentation method [29]; Carbon [to calculate organic matter (OM = 1.724 × C]), nitrogen, phosphorus and potassium content, soil pH and salinity were also evaluated.

**Fig. 2:**
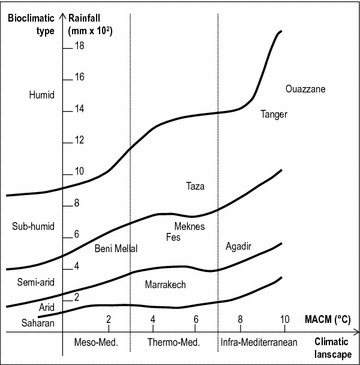
Bioclimatic diagram of the areas sampled [27]

Climatic typology of surveyed regions (Fig. 2) was characterized according to the modified Emberger diagram [30] consisting of annual rainfall and minimal average temperatures during the coldest month (MACM).

### Data analyses

Tukey’s range test was used to compare the frequency of *Meloidogyne* species between the regions (*P* value < 0.05). In order to assess the distribution of RKN in olive nurseries according to substrates, physicochemical characteristics and climate (Table 2), k + 1 multivariate method (MultiBlock Partial Least Squares (MBPLS) was followed and anlyzed in readxl, *ade*4 and R [31–33]. MBPLS regression is widely used for exploring and modelling relationships between several datasets to be predicted from several other datasets, and reveals contribution of each set in predicting response variables.

**Table 2:** Variables considered in the data analyses and corresponding codes

Variables	Code	Variables	Code
*Geographic region*		*Soil factors*	
Jbala	J	Coarse sand	cSa
Guerouane	G	Fine sand	fSa
Haouz	H	Coarse silt	cSi
Souss	S	Fine silt	fSi
		Clay	Cla
*Climate*		Organic matter	OM
Minimum temperature	MACM	Nitrogen	N
Annual rainfall	AR	Phosphorus	P
		Potassium	K
*Main substrate origin*		pH	pH
Crop topsoil	Crop	Conductivity	Con
Forest	Forest		
Riverbank	River		

## Results

*Meloidogyne* spp. specimens were detected in 52.1% of the nursery plants and in 23.2% of the orchard soil samples with a population range of 20–4000 individuals per dm^3^ of soil. All the RKN isolates reproduced on susceptible tomato plants, except one isolate detected in a traditional orchard from the Haouz region (isolate no 255).

### Species characterisation

**Fig. 3:**
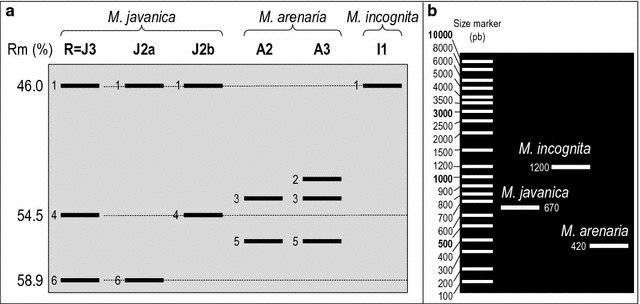
Biochemical and molecular patterns of *Meloidogyne* populations collected from cultivated olive soils in Morocco. **a** Species-specific esterase phenotypes detected (R: *M. javanica* reference population; Rm: relative migration; 1–6: number of specific bands according to *M. javanica* reference population). **b** Species-specific SCAR phenotypes detected

*M. javanica* reference population was confirmed as a J3 Est phenotype [22] with three bands (Rm of 46, 54.5 and 58.9%). Six phenotypes were detected among tested females (Fig. 3a and Table 3), labelling six different Est bands. J3 phenotype was detected widespread in both nurseries and orchards. Two phenotypes of *M. javanica* J2a (Rm of 46 and 58.9%) and J2b (Rm of 46 and 54.5%) were detected as being mixed with J3 in orchard isolates 260 and 252, respectively. One I1 Est phenotype, specific to *M. incognita* (Rm of 46%), was detected only as being mixed with J3 in low proportions of Souss and Haouz nurseries. Two a Est phenotypes specific to *M. arenaria* such as A2 (Rm of 53.75 and 56.25%) and A3 (Rm of 51, 53.75 and 56.25%) occurred as mixed populations in the Jabla nursery. These Est patterns were confirmed with species-specific SCAR patterns (Fig. 3b and Table 3).

**Table 3:** Biochemical and molecular diagnostics of the *Meloidogyne* species associated with olive trees in Morocco

Geographic region	Nursery	Patterns		Validated species (occurrence%)
		Est	SCARs	
Nurseries				
Souss	1	*Javanica* J3	*Javanica*	*M. javanica* (100%)
	2	*Incognita* I1/*Javanica* J3	*Incognita*/*Javanica*	*M. incognita* (20%) + *M. javanica* (80%)
	3	*Incognita* I1/*Javanica* J3	*Incognita*/*Javanica*	*M. incognita* (16%) + *M. javanica* (84%)
	4	*Incognita* I1/*Javanica* J3	*Incognita*/*Javanica*	*M. incognita* (10%) + *M. javanica* (90%)
	5	*Incognita* I1/*Javanica* J3	*Incognita*/*Javanica*	*M. incognita* (27%) + *M. javanica* (73%)
	6	*Javanica* J3	*Javanica*	*M. javanica* (100%)
	7	*Javanica* J3	*Javanica*	*M. javanica* (100%)
	8	*Incognita* I1/*Javanica* J3	*Incognita*/*Javanica*	*M. incognita* (13%) + *M. javanica* (87%)
	9	*Incognita* I1/*Javanica* J3	*Incognita*/*Javanica*	*M. incognita* (22%) + *M. javanica* (78%)
	10	*Javanica* J3	*Javanica*	*M. javanica* (100%)
	11	*Incognita* I1/*Javanica* J3	*Incognita*/*Javanica*	*M. incognita* (4%) + *M. javanica* (96%)
Haouz	12	*Javanica* J3	*Javanica*	*M. javanica* (100%)
	13	*Incognita* I1/*Javanica* J3	*Incognita*/*Javanica*	*M. incognita* (10%) + *M. javanica* (90%)
	14	*Javanica* J3	*Javanica*	*M. javanica* (100%)
	15	*Incognita* I1/*Javanica* J3	*Incognita*/*Javanica*	*M. incognita* (3%) + *M. javanica* (97%)
	16	*Incognita* I1/*Javanica* J3	*Incognita*/*Javanica*	*M. incognita* (7%) + *M. javanica* (93%)
	17	*Javanica* J3	*Javanica*	*M. javanica* (100%)
	18	*Incognita* I1/*Javanica* J3	*Incognita*/*Javanica*	*M. incognita* (8%) + *M. javanica* (92%)
	19	*Javanica* J3	*Javanica*	*M. javanica* (100%)
	20	*Javanica* J3	*Javanica*	*M. javanica* (100%)
Guerouane	21	*Javanica* J3	*Javanica*	*M. javanica* (100%)
	22	*Javanica* J3	*Javanica*	*M. javanica* (100%)
	23	*Javanica* J3	*Javanica*	*M. javanica* (100%)
	24	*Javanica* J3	*Javanica*	*M. javanica* (100%)
Jbala	25	*Arenaria* A2/*Arenaria* A3	*Arenaria*	*M. arenaria* (A2 = 75%; A3 = 25%)
Orchards				
Souss	252	*Javanica* J2b/*Javanica* J3	*Javanica*	*M. javanica* (J2 = 25%; J3 = 75%)
	253	*Javanica* J3	*Javanica*	*M. javanica* (100%)
Haouz	255	No multiplication on tomato	*Meloidogyne* spp.	
	258	*Javanica* J3	*Javanica*	*M. javanica* (100%)
	259	*Javanica* J3	*Javanica*	*M. javanica* (100%)
	260	*Javanica* J2a/*Javanica* J3	*Javanica*	*M. javanica* (J2 = 33%; J3 = 66%)
	261	*Javanica* J3	*Javanica*	*M. javanica* (100%)
	262	*Javanica* J3	*Javanica*	*M. javanica* (100%)
	285	*Javanica* J3	*Javanica*	*M. javanica* (100%)
	383	*Javanica* J3	*Javanica*	*M. javanica* (100%)
Tadla	308	*Javanica* J3	*Javanica*	*M. javanica* (100%)
	309	*Javanica* J3	*Javanica*	*M. javanica* (100%)
Guerouane	410	*Javanica* J3	*Javanica*	*M. javanica* (100%)

*Est* esterase isozyme phenotype, *SCAR* sequence characterised amplified regions

### Impact of environmental factors on *Meloidogyne* species distribution in nurseries

**Fig. 4:**
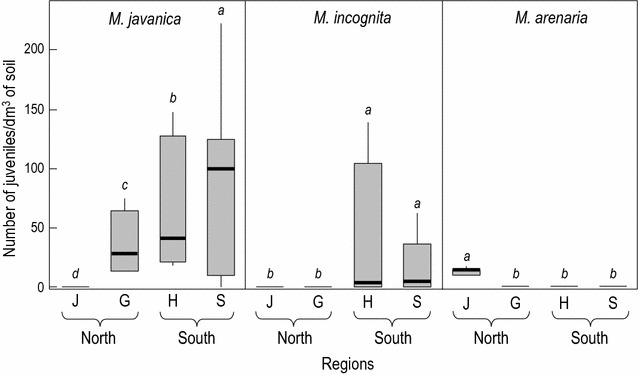
Boxplots for *Meloidogyne* species in the olive nurseries sampled according to the regions surveyed (*J* Jbala,* G* Guerouane;* H* Haouz;* S* Souss) (*a*–*d* indicates significant groups, *P* < 0.05)

**Fig. 5:**
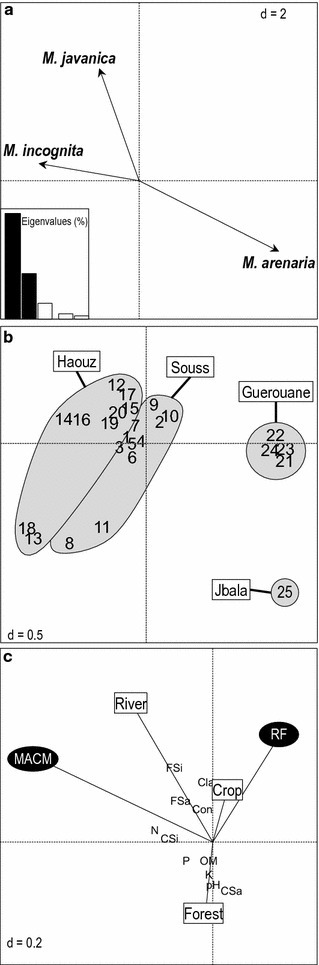
Multiblock analysis between the abundance of the *Meloidogyne* species and the substrate origins and the environmental factors in the olive nurseries. **a** PCA loading plot of the abundance of the *Meloidogyne* species; **b** score plot for the olive samples. **c** PCA loading plot of the environmental factors

Analysis of variance (Fig. 4) showed that RKN populations were more abundant in the southern regions (Souss and Haouz) as compared to the northern regions (Guerouane and Jbala). Genetic diversity of the olive trees in nurseries did not have any effect on the RKN distribution within and between nurseries (data not shown). Considering the other environmental variables (climate, physico-chemical characteristics and habitat origin of the substrates), the MBPLS analysis (Fig. 5a) clearly indicated opposite contributions of *M. incognita*, *M. javanica* and *M. arenaria*. It also confirmed (Fig. 5b) that *M. arenaria* was associated with the nursery surveyed in the Jbala region, while *M. javanica* and *M. incognita* were found in the Souss and Haouz nurseries. Nurseries surveyed in the Guerouane region and affected by high rainfall (RF, Fig. 5c) were clearly identified as being free of *M. incognita* (Fig. 4, Table 3). *M. incognita* was found to be associated with the nurseries of southern regions (Souss and Haouz), characterised by a higher MACM. Habitat origin of the substrates contributed less to the MBPLS analysis as compared to climate (Fig. 5c, second axis). However, it was clearly established that *M. javanica*, widespread in nearly all nurseries (96%, Table 3), was primarily associated with substrates prepared with large amounts of riverbank soils in the Haouz region and of crop soils in the Souss region. Physio-chemical soil factors did not greatly contribute to the structuring of *Meloidogyne* diversity.

### Distribution of the *Meloidogyne* species in nurseries and orchards

**Fig. 6:**
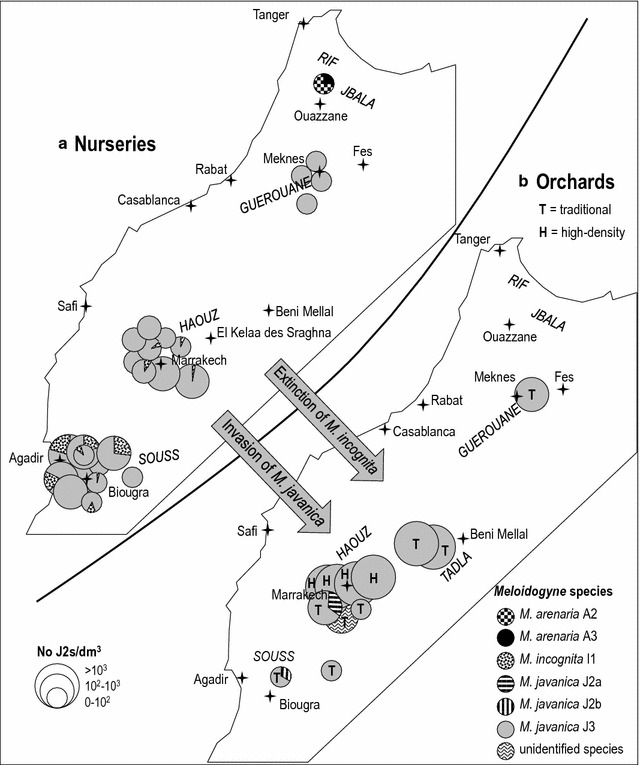
Spatial distribution and abundance of the *Meloidogyne* species identified in nurseries (**a**) and orchards (**b**)

Biochemical and molecular diagnoses confirmed the occurrence of *Meloidogyne* populations on cultivated olives in Morocco. *M. javanica* phenotype J3 was detected in 96% of the nurseries and in 92% of the orchards (Table 3), either traditionally or high-density cultivated, and was widespread throughout the main olive producing areas (Fig. 6). Two phenotypes of *M. javanica* J2a and J2b were detected only in traditional orchards as being mixed with J3 in the Haouz and the Souss regions, respectively (Fig. 6b). *M. incognita* phenotype I1 was detected only in nurseries and as mixed in low proportions with J3 (Fig. 6a, Table 3). Two *M. arenaria* phenotypes A2 and A3 occurred as mixed populations in the nursery of Jbala region (Fig. 6a, Table 3). *M. javanica* populations were more frequently abundant in the southern nurseries (up to 10^3^ juveniles/dm^3^ of soil in the Souss and Haouz regions) as compared to northern nurseries (less than 10^2^ juveniles in the Guerouane region). They were, however, less common in the Souss orchards than elsewhere (Fig. 6b). *M. incognita* populations flourished more in the Souss nurseries than in Haouz (with 16 and 4.5% of the total RKN populations, respectively), and the population of *M. arenaria* in Jbala nursery was low (< 10^2^ juveniles/dm^3^ of soil).

## Discussion

Main objective of this study was to understand the dispersal process of RKN from nurseries to orchards. RKN were detected in 1/2 of the nurseries and in less than 1/4 of the surveyed orchards. This corroborates with other reports which reveal only scarce detection of RKN in olive-producing areas [16]. Potential damage of these species to olive has never been properly investigated. However, *Meloidogyne* spp. are major pest of olive trees as high occurrence is usually noticed [34]. They induce considerable damage in nurseries and reduce olive growth in orchards [18, 19]. RKN population thresholds to olive plantlets are unknown, yet the population densities noticed during this study (up to 10^3^ juveniles/dm^3^ of soil) may present a potential risk to olive plantlets, in nurseries and fields. *M. incognita* and *M. javanica* significantly reduce shoot growth of olive cultivars in nurseries, implying a potential impact of RKN [18]. RKN damage in the irrigated sandy soils of the Souss and Haouz regions (high temperature and moisture) can be even higher as many new olive plantations have been established in context of the Moroccan Green Plan [35, 36].

*Meloidogyne* species were characterised through biochemical (PAGE esterase phenotypes) and molecular RAPD primer (SCAR) methods [16, 21]. Est phenotype is the most instructive biochemical identification technique for *Meloidogyne* species [37, 38] because of its species-specificity within the *Meloidogyne* genus [39–41]. All the phenotypes have previously been reported on other crops [22, 42] including olive [15, 16]. Nevertheless, some variability within *M. javanica* populations in orchards and within *M*. *arenaria* populations in northern nurseries was spotted. The phenotype J2a was already reported [43] whereas other phenotype J2b was previously diagnosed on peanut [44]. Phenotypes A2 and A3 [39, 45] were apparently clustered by geographic origin. Biochemical diagnostics were further confirmed by the molecular approach with SCAR markers that demonstrated their specificity to *M. incognita, M. arenaria* and *M. javanica* [46].

*M. arenaria, M. incognita* and *M. javanica* have previously been reported on olive trees in the Mediterranean basin, Asia and South America [47]. It is suggested that these species have the same geographic distribution on various hosts [23]. *M. javanica* and *M. incognita* have been reported as the most common species in the olive nurseries of Spain [18], but *M. incognita* was not detected in orchards. Widespread distribution of *M. javanica* was noticed in the southern regions (Souss and Haouz), known for RKN-susceptible vegetable production. *M. javanic*–*M. incognita* distribution in Moroccan olive nurseries is in line with their distribution in Iran [48] and Spain [18] where more than 20% of olive plantlets were infested by *M. javanica* alone, and 10% were co-infected by *M. javanica* and *M. incognita*.

Distribution of these RKN species in nurseries could be related to human activities and favourable environmental factors. As in orchards, intensive nursery monoculture is very susceptible to build-up of nematode populations and ultimately damaging the tree seedlings. High temperature is a favourable factor for the reproduction and multiplication of *M. javanica* and *M. incognita* populations. In fact, temperature is the key feature for their survival and fecundity [49]. High temperatures also favour hatching, mobility and root invasion of *M. javanica*. There are reports from southern Spain and northern Iran revealing alarming *Meloidogyne* population densities (28.6 and 22.3% yield loss, respectively) in nurseries [18, 48]. Besides, *M. arenaria*, usually found in tropical, subtropical, temperate mild regions and in glasshouses under cooler climates [50], was found in the coldest regions of northern Morocco with high annual rainfall.

In short, *M. javanica* and *M. incognita* were most probably introduced into nurseries through soil substrates from agricultural fields and riverbanks. Their widespread distribution in nurseries with highly infested substrates confirmed high fitness properties. It clearly confirms that their adaptation and reproductive success is mainly due to their mitotic parthenogenetic reproduction [20] and ability to infect various plant species [51]. *M. arenaria* was only found in the northern nursery (Jbala region), grown on forest soil substrates. It can be linked to previous reports about phenotype A2 on wild olives [16].

Introduction of RKN in orchards after the transplantation of rooted plantlets, seems obvious, as in case of endoparasitic nematodes. Soil nematodes move very short distances so their long-distance dissemination is only possible through human activities [52]. Consequently, their widespread distribution in cultivated olive-growing areas might have been inducted from nurseries [53]. Therefore, in case of olive production systems, the invasive species status can be attributed to *M. javanica* [54], which out-competes native species especially in high-density orchards [55]. However, selection processes might have occurred as no *M. incognita* was detected in orchards despite their occurrence in nurseries. This extinction could be explained either by non-adequate life conditions (no fitted niches) or competition during long-term cohabitation with *M. javanica* or with native PPN species. Species selection after invasion might influence the capacity to disperse [56] along with the physiological tolerance to the new environment [57]. Presence of *M. arenaria* only in one nursery supports the hypothesis of refuge conditions of the area for this species [58], especially in wild olive [59], and that could explain why *M. arenaria* did not disperse in a context of the low human activity.

## Conclusion

To conclude, introductions of pest species through cropping practices are usually irreversible and frequently cause undesirable impacts. Therefore, we can assume that olive production systems are at greater PPN invasion risks. Sanitisation of the nursery substrates is mandatory to avoid nematode problems in new plantations.

## Abbreviations

DNA: deoxyribonucleic acid
Est: esterase
F/R: forward/reverse
ISO: International Organization for Standardisation
MACM: minimal average temperatures of the coldest month
MBPLS: MultiBlock Partial Least Squares
OM: organic matter
PAGE: polyacrylamide gel electrophoresis
PCA: principal component analysis
PCR: polymerase chain reaction
PPN: plant-parasitic nematodes
RAPD: random amplification of polymorphic DNA
RKN: root-knot nematodes
SCAR: Sequence Characterized Amplified Region
